# Characteristic Gene Selection via Weighting Principal Components by Singular Values

**DOI:** 10.1371/journal.pone.0038873

**Published:** 2012-07-10

**Authors:** Jin-Xing Liu, Yong Xu, Chun-Hou Zheng, Yi Wang, Jing-Yu Yang

**Affiliations:** 1 Bio-Computing Research Center, Shenzhen Graduate School, Harbin Institute of Technology, Shenzhen, Guangdong, China; 2 College of Electrical Engineering and Automation, Anhui University, Hefei, Anhui, China; 3 School of Mechanical Engineering and Automation, Shenzhen Graduate School, Harbin Institute of Technology, Shenzhen, Guangdong, China; 4 School of Computer Science and Technology, Nanjing University of Science and Technology, Nanjing, Jiangsu, China; 5 College of Information and Communication Technology, Qufu Normal University, Rizhao, Shandong, China; 6 Key Laboratory of Network Oriented Intelligent Computation, Shenzhen Graduate School, Harbin Institute of Technology, Shenzhen, Guangdong, China; Institution of Automation, CAS, China

## Abstract

Conventional gene selection methods based on principal component analysis (PCA) use only the first principal component (PC) of PCA or sparse PCA to select characteristic genes. These methods indeed assume that the first PC plays a dominant role in gene selection. However, in a number of cases this assumption is not satisfied, so the conventional PCA-based methods usually provide poor selection results. In order to improve the performance of the PCA-based gene selection method, we put forward the gene selection method via weighting PCs by singular values (WPCS). Because different PCs have different importance, the singular values are exploited as the weights to represent the influence on gene selection of different PCs. The ROC curves and AUC statistics on artificial data show that our method outperforms the state-of-the-art methods. Moreover, experimental results on real gene expression data sets show that our method can extract more characteristic genes in response to abiotic stresses than conventional gene selection methods.

## Introduction

The growth of plants is greatly affected by a variety of abiotic stresses, such as cold, drought, salt, heat, UV-B light, osmotic press, and so on. In response to these abiotic stresses, plants have evolved a number of defense mechanisms that can increase tolerance to the adverse conditions. The underlying concept is that there exists a specific set of interacting genes responding to the abiotic stresses. Therefore, understanding abiotic stress responses is now thought to be one of the most important topics in plant science [1].

In order to obtain the characteristic genes responding to these stresses, many conventional experimental methods were proposed, such as RT-PCR [2], [3] and Northern blotting [4], [5], etc. RT-PCR can accurately position the genes in tissue or cell and Northern blotting can display the information of detected genes. However, these two methods have the following fatal flaw: only a limited amount of genes can be simultaneously studied. To overcome this flaw, people have developed the gene microarray technology [6]–[8], which makes it possible to monitor gene expression levels on a genomic scale [9], [10].

With the rapidly development of gene microarray technology, how to efficiently analyze gene expression data becomes a matter of great urgency. During the last decade, feature selection from gene expression data has been extensively studied. The most commonly used methods of feature selection first calculate a score for each gene, respectively, then select the genes with high scores [11]. These methods are often denoted as univariate feature selections (UFS). The main virtues of UFS are: (a) intuitive and easy to understand; (b) computationally simple; and (c) fast [12]. A common disadvantage of UFS based methods is that each feature is separately considered, thereby ignoring feature dependencies. In order to handle the problem, the method of multivariate feature selection (MFS), also denoted as dimension reduction, was introduced [13]. MFS uses all the gene expression data simultaneously to select the genes. Until now, many mathematical methods for MFS have been used for gene expression data analysis. For example, Park *et al.* gave the theoretical analysis on feature extraction capability of class-augmented PCA [14]. Ma *et al.* used PCA to identify differential gene pathways in [15]. De Haan *et al.* used PCA to analyze microarray data [16]. Musumarra *et al.* used PLS to identify genes for new diagnostics [17]. Boulesteix *et al*. provided a systematic comparison of the PLS methods for the analysis of gene data [18].

**Figure 1:**
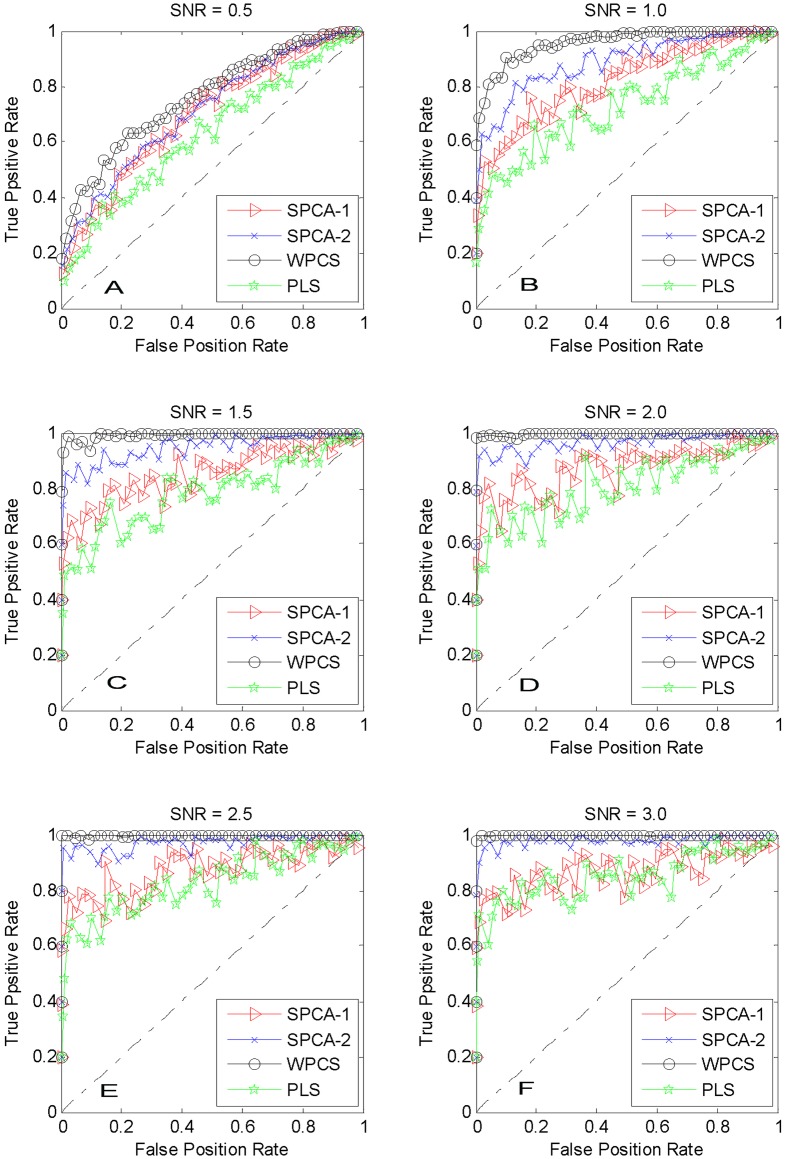
ROC curves for artificial data. (SNR denotes the signal-to-noise ratio).

However, the classical methods, such as PCA and PLS, still have some drawbacks, e.g. the principal components (PCs) of PCA or the latent components (LCs) of PLS are usually dense, which makes it difficult to interpret PCs or LCs without subjective judgment. To overcome these drawbacks, many mathematical tools have been devised to reduce the complexity of the data. Among them, sparse methods have significant advantage, while giving up little statistical efficiency. For example, Zou *et al*. proposed sparse PCA using the lasso [19]. In [20], Journée *et al*. described a general power method for sparse PCA. Lai *et al*. used sparse local discriminant projections for feature extraction [21]. Moreover, many sparse methods have been widely used for gene expression data analysis. Lass *et al*. used the SPCA for clustering and feature selection [22]. In [23], Witten *et al*. proposed a penalized matrix decomposition, which was used to analyze plant gene expression data by Liu *et al*. [24]. Cao *et al*. used sparse PLS discriminant analysis for biologically relevant feature selection [25].

**Table 1:** AUC statistics for artificial data.

	SNR = 0.5	SNR = 1	SNR = 1.5	SNR = 2	SNR = 2.5	SNR = 3
SPCA-1	0.6985000	0.8186722	0.8517611	0.8738500	0.8733944	0.8665222
SPCA-2	0.7131000	0.8888778	0.9469222	0.9668333	0.9730667	0.9800500
WPCS	0.7615000	0.9579889	0.9918333	0.9971667	0.9988222	0.9989444
PLS	0.6282000	0.7269556	0.7812667	0.8061889	0.8261889	0.8506667

Though sparse methods are useful, yet these methods used the first LC of PLS or PC of SPCA to select feature. For example, Boulesteix *et al*. used the first LC of PLS to select the important genes [18]. Liu *et al*. used the first PC of SPCA for characteristic genes selection [26]. These methods indeed assumed that the first component of PLS or SPCA plays a dominant role in gene selection. However, we identify that in a number of cases this assumption is not satisfied and conventional PCA-based gene selection methods usually provide poor results. Actually, not only the first LC or PC but also the remaining LCs or PCs include the important information for gene selection [27]. So if only the first LC or PC is used in selecting genes, the poor results may have been obtained with loss of some important information.

In this paper, in order to select characteristic genes, a novel method is proposed that is based on the weighted PCs of SPCA by singular values (WPCS). First, the PCs of SPCA and singular values are calculated. Second, using the singular values as the weights of PCs, the weighted PCs (WPC) are gotten. Then, as the absolute value of the *i*-th entry of every WPC somewhat denotes the importance of the *i*-th gene, we take the sum of all the *i*-th entries of all the WPC as the extent of importance of the *i*-th gene and use it to select features. The genes corresponding to the first 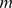 largest sums are selected as characteristic genes. The experimental results show that our method is efficient and powerful for gene selection. Our work has the following contributions: first, it proposes, for the first time, the method of weighting PCs by singular values for gene selection. Second, from the viewpoint of minimizing reconstitution error, it clearly presents the idea of the proposed method. Third, it conducts a large number of gene selection experiments.

**Figure 2:**
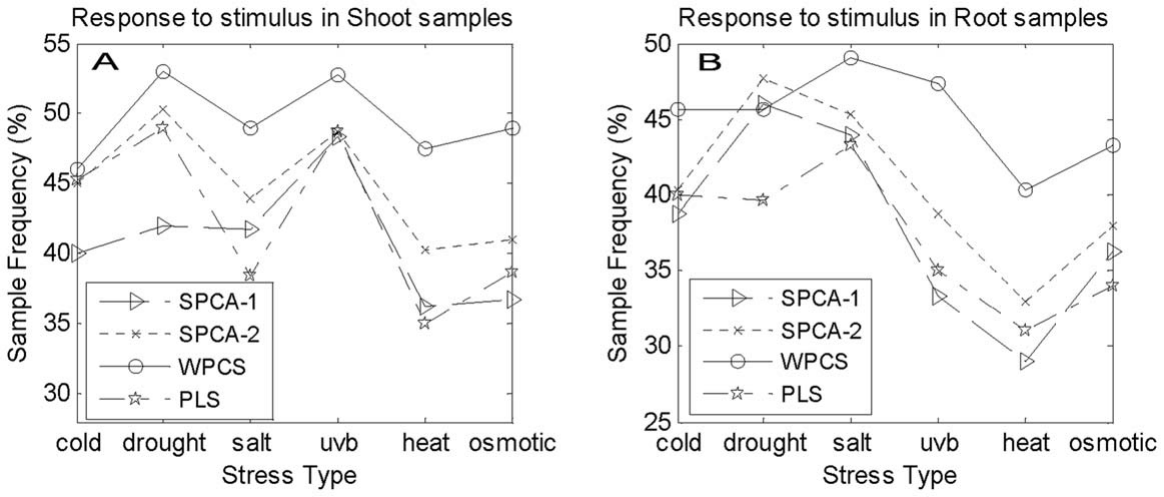
The Response to stimulus (GO: 0050896).

## Results and Discussion

In this section, our proposed WPCS method is compared with the following existing methods. (a) SPCA-1 method uses only the first one PC of SPCA (proposed by Journée *et al.* [20]) to identify the characteristic genes; (b) SPCA-2 method uses the first two PCs of SPCA to identify the characteristic genes; (c) PLS method uses partial least squares regression (PLS) (proposed by Boulesteix *et al.* [18]) to identify the characteristic genes.

**Figure 3:**
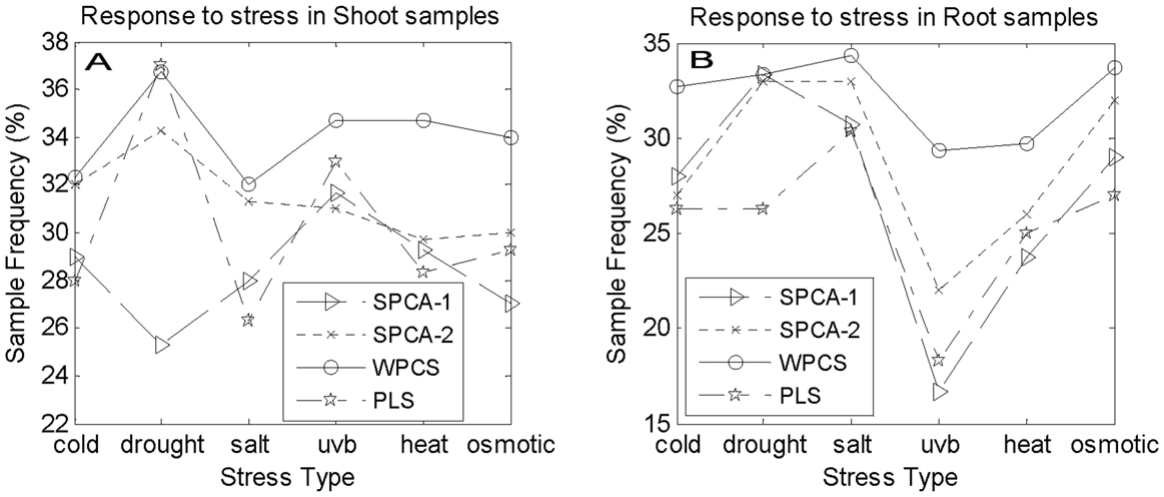
The Response to stress (GO: 0006950).

First, these methods are carried on the artificial data. Then, these methods are used to extract the characteristic genes responding to abiotic stresses from real gene expression data.

### Simulation on Artificial Data

To investigate the performance of the methods, the average receiver operator characteristic (ROC) curves are shown in Figure 1 with six different SNR.

**Table 2:** Response to stimulus (GO: 0050896) in shoot samples.

Stress	SPCA-1		SPCA-2		WPCS		PLS	
Type	SF♀	PV	SF	PV	SF	PV	SF	PV
Cold	120/300* 40.0%	2.39E-21	135/300 45.0%	2.19E-30	**138/300 46.0%**	2.33E-32	136/300 45.3%	5.19E-31
Drought	126/300 42.0%	8.50E-25	151/300 50.3%	1.62E-41	**159/300 53.0%**	1.17E-47	147/300 49.0%	2.36E-38
Salt	125/300 41.7%	3.27E-24	132/300 44.0%	1.19E-28	**147/300 49.0%**	8.34E-39	115/300 38.5%	8.57E-19
UV-B	145/300 48.3%	6.44E-37	146/300 48.7%	1.31E-37	**158/300 52.7%**	7.51E-47	146/300 48.7%	1.20E-37
Heat	109/300 36.3%	2.05E-15	121/300 40.3%	7.12E-22	**142/300 47.5%**	2.89E-35	105/300 35.0%	1.24E-13
Osmotic	110/30036.7%	6.18E-16	123/300 41.0%	7.06E-23	**147/300 49.0%**	2.19E-38	116/300 38.7%	4.96E-19

Note: The response to stimulus on characteristic genes are shown, whose background frequency in TAIR set is 4570/29887 (15.3%), where 4570/29887 denotes having 4570 genes to respond to stimulus in whole 29887 genes set.

♀ SF: sample frequency, PV: P-value.

* In the table, the sample frequency, e.g. 120/300, denotes the method select 300 genes, in which there are 120 genes responding to stimulus.

**Table 3:** Response to stimulus (GO: 0050896) in root samples.

Stress	SPCA-1		SPCA-2		WPCS		PLS	
Type	SF	PV	SF	PV	SF	PV	SF	PV
Cold	116/300 38.7%	4.79E-19	121/300 40.3%	1.12E-21	**137/300 45.7%**	1.78E-31	120/300 40.0%	3.46E-21
Drought	138/300 46.0%	3.62E-32	**143/300 47.7%**	1.48E-35	137/300 45.7%	1.55E-31	119/300 39.7%	1.12E-20
Salt	132/300 44.0%	2.52E-28	136/300 45.3%	6.80E-31	**147/300 49.0%**	2.06E-38	130/300 43.3%	4.23E-27
UV-B	100/300 33.3%	2.60E-11	116/300 38.7%	4.79E-19	**142/300 47.3%**	6.67E-35	105/300 35.0%	1.34E-13
Heat	87/300 29.0%	3.30E-06	99/300 33.0%	4.75E-11	**121/300 40.3%**	6.93E-22	93/300 31.0%	1.41E-08
Osmotic	109/300 36.3%	1.92E-15	114/300 38.0%	5.57E-18	**130/300 43.3%**	4.43E-27	102/300 34.0%	3.29E-12

**Table 4:** Response to stress (GO: 0006950) in shoot samples.

Stress	SPCA-1		SPCA-2		WPCS		PLS	
Type	SF	PV	SF	PV	SF	PV	SF	PV
Cold	87/300 29.0%	5.19E-25	96/300 32.0%	1.52E-31	**97/300 32.3%**	2.61E-32	84/300 28.0%	6.72E-23
Drought	76/300 25.3%	9.02E-18	103/300 34.3%	5.07E-37	110/300 36.7%	7.81E-43	**111/300 37.0%**	1.68E-43
Salt	84/300 28.0%	6.06E-23	94/300 31.3%	3.57E-30	**96/300 32.0%**	1.15E-31	79/300 26.3%	8.58E-20
UV-B	95/300 31.7%	1.28E-30	93/300 31.0%	4.13E-29	**104/300 34.7%**	8.09E-38	99/300 33.0%	1.07E-33
Heat	88/300 29.3%	1.46E-25	89/300 29.7%	2.20E-26	**104/300 34.7%**	5.61E-38	85/300 28.3%	1.31E-23
Osmotic	81/300 27.0%	7.48E-21	90/300 30.0%	5.38E-27	**102/300 34.0%**	4.37E-36	88/300 29.3%	1.32E-25

Note: The response to stress (GO: 0006950) obtained by GO Term Enrichment Analysis are shown, whose background frequency in TAIR set is 2351/29887 (7.9%), where 2351/29887 denotes having 2351 genes to respond to stress in whole 29887 genes set.

**Table 5:** Response to stress (GO: 0006950) in root samples.

Stress	SPCA-1		SPCA-2		WPCS		PLS	
Type	SF	PV	SF	PV	SF	PV	SF	PV
Cold	84/300 28.0%	7.21E-23	81/300 27.0%	9.01E-21	**98/300 32.7%**	6.51E-33	79/300 26.3%	1.48E-19
Drought	100/300 33.3%	1.68E-34	99/300 33.0%	1.03E-33	**100/300 33.3%**	1.58E-34	79/300 26.3%	1.34E-19
Salt	92/300 30.7%	1.79E-28	99/300 33.0%	9.23E-34	**103/300 34.3%**	6.50E-37	91/300 30.3%	9.12E-28
UV-B	50/300 16.7%	1.30E-04	66/300 22.0%	5.55E-12	**88/300 29.3%**	1.33E-25	55/300 18.3%	1.09E-06
Heat	71/300 23.7%	1.11E-14	78/300 26.0%	4.21E-19	**89/300 29.7%**	2.14E-26	75/300 25.0%	3.20E-17
Osmotic	87/300 29.0%	6.81E-25	96/300 32.0%	1.89E-31	**101/300 33.7%**	2.52E-35	81/300 27.0%	6.93E-21

Figure 1(A) and 1(B) show that our WPCS and the competitive methods can identify the patterns even with very low SNR. As shown in Figure 1, with different SNR, WPCS outperforms other methods. For example, with SNR = 1.0, PLS method are dominated by SPCA-2 and SPCA-1; and our WPCS can achieve the best results.

**Table 6:** The numbers of response to cold (GO: 0009410) in shoot samples.

Method	SPCA-1	SPCA-2	WPCS	PLS
Number and percent	38 genes,12.7%	45 genes,15.0%	48 genes,16.0%	33 genes,11.0%
P-value	1.45E-27	2.43E-36	2.73E-40	1.18E-21

The area under curve (AUC) statistics are listed in Table 1, from which we can conclude that under the same SNR, the ascending order of accuracy given by these methods is: PLS, SPCA-1, SPCA-2 and WPCS.

**Table 7:** Different genes of response to cold (GO: 0009410) in shoot samples.

Gene No.	Function of Gene
At1g21910	Participates in plant developmental processes as well as biotic and/or abiotic stress signaling.
At1g22770	Regulates several developmental processes, such as circadian clock, carbohydrate metabolism, and cold stress response.
At1g29395	Expression is induced by short-term cold-treatment, water deprivation, and abscisic acid (ABA) treatment.
At2g19450	Role in senescence and seed development induced by cold-stress.
At2g25930	Temperature stress reduced the pyk20 transcript level.
At2g28900	Predominantly expressed in leaves and is also inducible by cold treatment.
At2g33380	Plays a role as a peroxygenase involved in oxylipin metabolism during biotic and abiotic stress.
At2g38470	Involved in response to various abiotic stresses
At2g47180	Increases tolerance to chilling stress
At3g05880	Induced by low temperatures, dehydration and salt stress.
At3g48360	Mediates multiple responses to nutrients, stresses, and hormones.
At3g53990	Low temperature and salt responsive protein family.
At4g30650	Low temperature and salt expression protein homologous.
At4g30660	Putative low temperature and salt responsive protein.
At4g37610	Under cold stress indicates increased expression.
At5g52300	Induced by low temperature, exogenous abscisic acid (ABA) and drought.
At5g57560	Controlling tolerance to cold stress

From the experiments on artificial data, a conclusion can be drawn that WPCS method outperforms other methods for feature selection.

**Table 8:** The numbers of response to light stimulus (GO: 0009416) in root samples.

Method	SPCA-1	SPCA-2	WPCS	PLS
Number and percent	17 genes, 5.7%	20 genes, 6.7%	24 genes, 8.0%	17 genes, 5.7%
P-value	1.74E-02	2.90E-04	7.42E-07	1.55E-02

### Gene Ontology (GO) Analysis

The Gene Ontology (GO) Term Enrichment tool can be used to help discover what those genes may have in common [28]. GOTermFinder is a web-based tool that finds the significant GO terms shared among a list of genes. The analysis of GOTermFinder provides significant information for the biological interpretation of high-throughput experiments. In this paper, our proposed method will be evaluated by GOTermFinder [29], which is publicly available at http://go.princeton.edu/cgi-bin/GOTermFinder. Its threshold parameters are set as following: maximum p-value = 0.01 and minimum number of gene products = 2.

**Table 9:** Different genes of response to light stimulus (GO:0009416) in root samples.

Gene No.	Function of Gene
At2g29500	HSP20-like chaperones superfamily protein.
At3g54890	Encodes a component of the light harvesting complex associated with photosystem I.
At3g55120	Catalyzes the conversion of chalcones into flavanones.
At5g02810	Acts as transcriptional repressor of CCA1 and LHY.
At5g12030	Encodes a cytosolic small heat shock protein with chaperone activity that is induced by heat and high light intensity stress.
At5g15960	stress-responsive protein (KIN1).
At5g24470	Encodes a pseudo-response regulator whose mutation affects various circadian-associated biological events such as red light sensitivity of seedlings during early photomorphogenesis.
At5g45340	abscisic acid 8′-hydroxylase 3.

Here, only the main results of GO are given. Figure 2 shows the sample frequency of response to stimulus (GO: 0050896) given by the four methods. From Figure 2(A), WPCS method outperforms the others in all the data sets of shoot samples with six different stresses. Figure 2(B) shows that only in drought-stress data set of root samples, our method is dominated by SPCA-1 and SPCA-2 methods. In other data sets, our method is superior to the others.

**Table 10:** The number of each stress type in the raw data.

Stress Type	cold	drought	salt	UV-B	heat	osmotic	control
Number	6	7	6	7	8	6	8

Figure 3(A) shows the sample frequency of response to stress (GO: 0006950) in shoot samples. From Figure 3(A), it can be seen that only in drought-stress data set, the PLS method is slightly superior to our method. In other data sets, our method is superior to the other methods. Figure 3(B) shows that only in drought-stress data set of root samples, our WPCS gives a similar result to that of SPCA-1 and SPCA-2 methods, and exceeds that of PLS method. In other data sets, our WPCS method surpasses the others.

**Figure 4:**
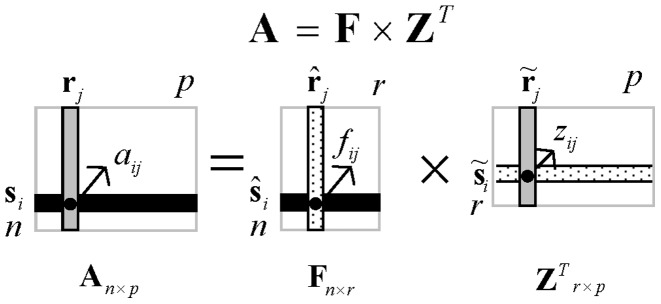
The graphical depiction of SPCA of a matrix A with factor scores 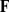 and PCs 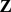. In this figure, with factor scores 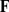 and PCs 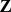. 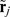 is the row vector of PCs 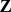 the *j*-th gene, which transforms the original data vector 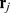 into factor scores 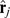. Correspondingly, 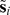 is the column vector of PCs 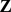, which transforms the original data vector 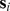 into factor scores 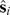.

The remarkable results are listed in Tables 2–5. The number of genes responding to stimulus (GO: 0050896) selected by the four methods in shoot and root samples are listed in Table 2 and Table 3, respectively.

As Table 2 listed, in shoot samples, WPCS method outperforms the others in all the data sets with six different stresses. As Table 3 listed, in root samples, only in drought-stress data set, WPCS method is dominated by SPCA-2. For other stresses data sets, WPCS outperforms our competitive methods.

**Figure 5:**
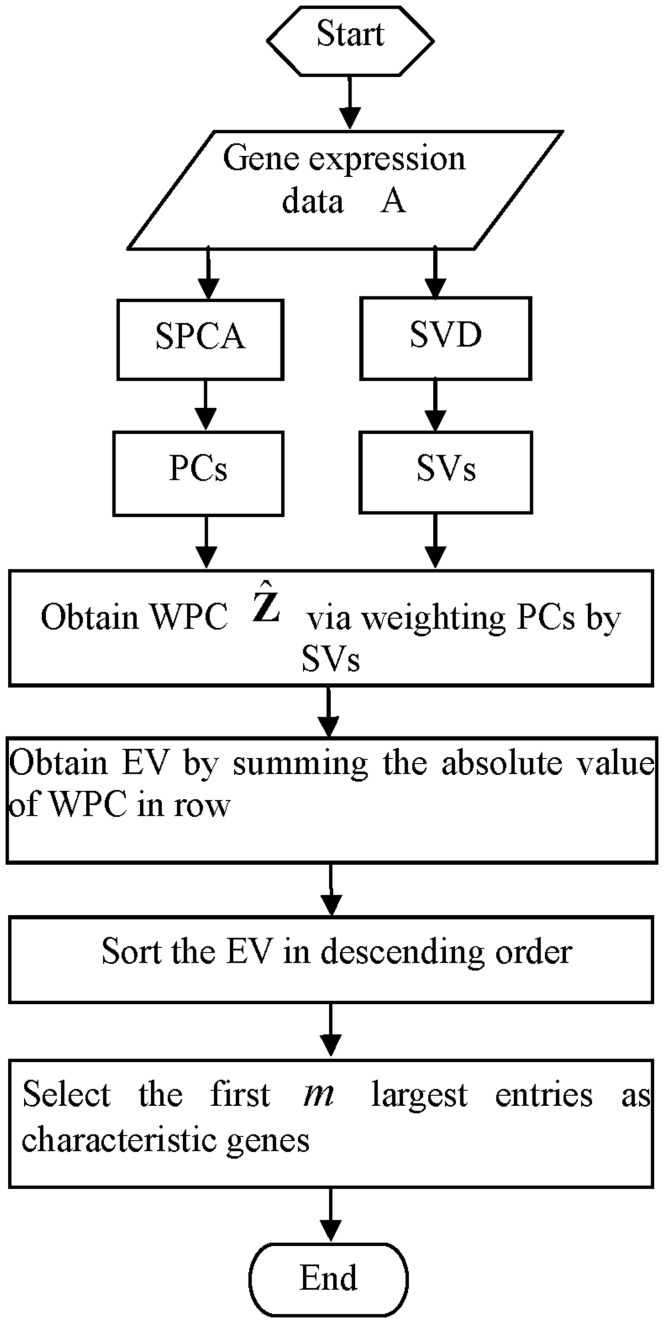
Workflow diagram of WPCS.

Table 4 and Table 5 give the gene numbers and P-value of response to stress (GO: 0006950) selected by the four methods in shoot and root samples, respectively.

To sum up, for all the data sets except drought-stress data set, our method is superior to other methods. For the drought-stress data set in shoot samples, only the PLS method slightly suppresses our WPCS method.

To further study the characteristic genes closely related to the stresses, the cold stress in shoot samples and UV-B stress in root samples are analyzed. Table 6 lists the numbers of response to cold (GO: 0009410) in shoot samples selected by these methods. The background sample frequency of response to cold (GO: 0009410) is 0.9% (276/29887). As Table 6 listed, our method can select more genes than others.

In detail, we compare the genes selected by WPCS with the genes selected using others. Different genes selected using WPCS and neglected by other methods are listed in Table 7. As Table 7 listed, the functions of genes selected using WPCS are closely related with cold stress.

Table 8 gives the numbers of response to light stimulus (GO: 0009416) in root samples selected using these methods. The background sample frequency of response to light stimulus (GO: 0009416) is 1.8% (547/29887).

As Table 8 listed, WPCS can select more genes than others. Moreover, we compare the genes selected by WPCS with the ones by other methods. The genes selected using WPCS and neglected by others are listed in Table 9. As Table 9 listed, the functions of genes selected using WPCS are closely related with UV-B stress.

From the experiments and analyses on gene expression data, a conclusion can be drawn that WPCS method is very efficient and powerful for gene selection.

### Conclusion

In this paper, a novel method of gene selection, WPCS, is proposed, that uses the weighted PCs by SVs as the basis of selection. The idea of WPCS is clearly shown. WPCS works as follows. First, it obtains the PCs of SPCA and SVs. Second, using the SVs as the weights of PCs, it obtains the WPC. Then, it sums the absolute value of the WPC in row, and sorts the sum in descending order. Finally, it selects the genes corresponding to the top part of the sum as the characteristic genes. A large number of experiments on artificial data and gene expression data demonstrate that the proposed WPCS method outperforms the state-of-the-art gene selection methods. For gene expression data, WPCS can extract more characteristic genes in response to abiotic stresses than the other methods.

## Materials and Methods

### Artificial Data

The artificial data are in 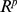 with 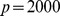 and generated as 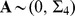. Let 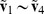 be four 2000-dimensional vectors, such that 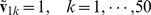, and 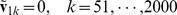; 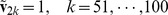, and 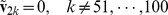; 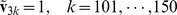, and 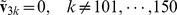; and 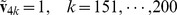, and 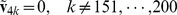. Let 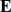 be 2000-dimensional noise matrix, 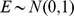. Then the noise matrix is added to 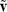 with different Signal-to-Noise Ratios (SNR). The first four eigenvectors of 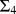 are chosen to be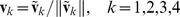. To make these four eigenvectors dominate, we let the eigenvalues be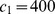,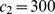, 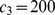, 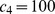 and 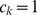 for 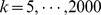. Then the simulation scheme in [30] is used to generate the artificial data, which include ten samples in each test.

### Gene Expression Data

The raw data include two classes: roots and shoots in each stress, which were downloaded from NASCArrays [http://affy.arabidopsis.info/] [31], reference numbers are: control, NASCArrays-137; cold stress, NASCArrays-138; osmotic stress, NASCArrays-139; salt stress, NASCArrays-140; drought stress, NASCArrays-141; UV-B light stress, NASCArrays-144; and heat stress, NASCArrays-146. The sample numbers of each stress type are listed in Table 10. There are 22810 genes in each sample. The data are adjusted for background of optical noise using the GC-RMA software by Wu *et al*. [32] and normalized using quantile normalization. The results of GC-RMA are gathered in a matrix for further processing.

### Selection of the Parameters

In SPCA, we take 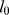-norm penalty and set 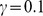. In PLS, only the first component is used. For the sake of comparison, on gene expression data, 300 genes are roughly selected by all the methods.

### Singular Value Decomposition (SVD)

In this subsection, the details of the WPCS method are presented. Let 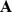 denote an 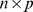 matrix of real-valued gene expression data, which consists of 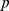 genes in 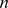 samples. In the case of gene expression data, 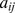 is the expression level of the *j*-th gene in the *i*-th sample. The elements of the *j*-th column in 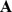 form the n-dimensional vector 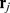, which is referred to as the transcriptional response of the *j*-th gene. Correspondingly, the elements of the *i*-th row in 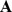 form the p-dimensional vector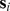, referred to as the expression profile of the *i*-th sample. Usually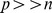, so it is a classical small-sample-size problem. To integrate SPCA and SV, the singular value decomposition (SVD) and the SPCA via cardinality penalty (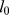-penalty) are introduced as follows.

If the variables contained in the columns of 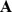 with rank 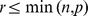 are centered, the equation for SVD of 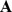 is as follows:
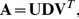
(1)where 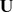 is an 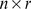 matrix of left singular vectors with 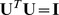, 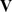 is a 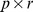 matrix of right singular vectors with 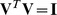, and 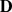 is a diagonal matrix of singular values. Let 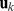 denote column 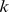-th of 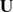, let 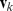 denote column 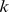-th of 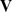, and note that 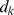 denotes the *k*-th diagonal element of the matrix 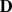. According to Eckart *et al.* [33],
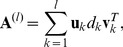
(2)which is the closest rank-l matrix to 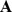. The term “closest” means that 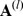 minimizes the square error sum between the elements of 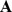 and 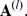


### Sparse Principal Component Analysis (SPCA)

The results given with 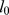
- and 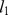
- norm penalty in SPCA are similar, which is also shown in [20]. Since the 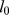-norm is faster than 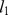-norm, 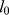-norm penalty is taken on SPCA. Let 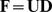 and 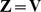, eq.(1) can be written as follows:
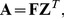
(3)where 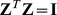 and 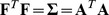. It is the classical PCA formation.

Extracting one principal component (PCs) amounts to computing the dominant eigenvector of 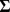 (or, equivalently, dominant right singular vector of 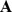). That is, PCA seeks to project the data onto the linear combination of variables that maximizes the sample variance. It is well-known that the solution to this problem is given by the right singular vector of 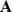. In general, PCs is not expected to have many zero coefficients. So, to makes it easy to interpret PCs without subjective judgment, Sparse PCA proposed by Journée *et al*. in [20] is used to generate the sparse PCs.

Let us consider the optimization problem.
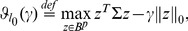
(4)with sparsity-controlling parameter 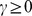, 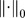 denotes the 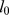-norm, that is, the number of non-zero components (cardinality).

According to [20], eq.(4) can be rewritten as follows:
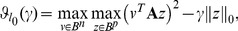
(5)where the maximization with respect to 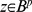 for a fixed 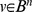 has the closed form solution



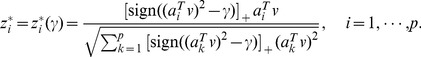
(6)According to [20], eq.(5) can be cast in the following form:
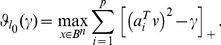
(7)


Here, for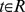, sign(t) denotes the sign of the argument and 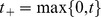.

For large enough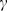, 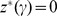. Since.
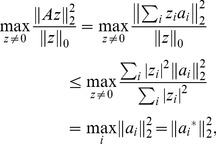
(8)we get.
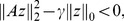
(9)for all nonzero vector 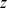, when 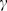 > 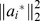.So, this derivation assumes that 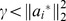, then.



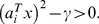
(10)Otherwise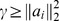, so
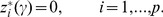
(11)


### The Idea of the Proposed Method

The residual sum of squares (RESS) can be used for evaluating the quality of the reconstitution of 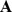 with 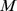 PCs of SPCA. According to [34], it can be computed as follows:
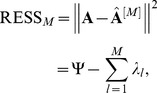
(12)where 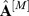 is a reconstitution of 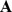, 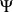 denotes the sum of all the squared elements, and 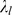 is the singular value of the *l*-th component. The smaller the value of RESS is, the better the SPCA model is. From eq.(12), we can see that a larger 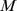 may give a better estimation of 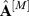. If 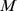 takes value 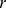 (rank of 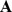),
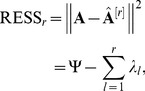
(13)where the matrix 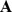 can be perfectly reconstituted. Let 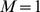, the 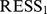 can be expressed as follows:
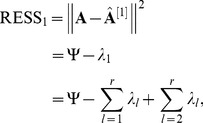
(14)Substituting eq.(13) into eq.(14), the 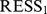 can be obtained as follows:



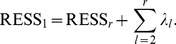
(15)As eq.(15) shown, if only one PC is used to reconstitute the matrix 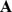, 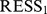 may be larger 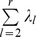 than 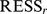. So, if only the first PC is used for characteristic gene selection, some important information may be lost, especially when the second one or two SVs are approximately equal to the first one. In order to obtain a better reconstitution, all the PCs of SPCA need to be utilized.

In SPCA, 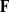 is the matrix of factor scores and 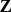 is a loading matrix of the principal components (PCs), which transforms the original data matrix into factor scores. The data matrix 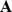, factor scores matrix 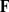 and PCs 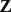 are shown in Figure 4.

As Figure 4 shown, the PCs 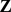 give the coefficients of the linear combinations used to compute the factors scores 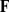. So the bigger the absolute value of the elements in PCs 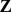 is, the more contribution it gives for the factor scores matrix, the more important the corresponding gene in 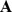 is. So the characteristic genes can be selected according to the PCs 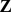.

Let.
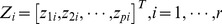
(16)denote the *i*-th PC, and then the PCs can be given as the follows:



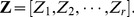
(17)Substituting 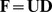 into eq.(3), it can be reformed as follows:
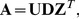
(18)where 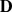 is the diagonal matrix of singular values. Let
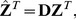
(19)eq.(18) can be reformed as follows:



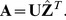
(20)Substituting eq.(17) into eq.(19),
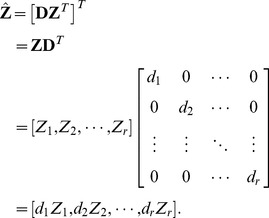
(21)


The matrix 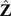 is referred to as weighted PCs (WPC), which can be obtained via weighting PCs by the diagonal matrix of SVs (WPCS).

Substituting eq.(16) into eq.(21), the WPC can be reformed as follows:
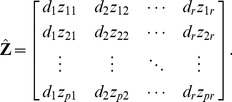
(22)


As the absolute value of the *i*-th row of WPC 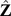 somewhat denotes the importance of the *i*-th gene, the absolute value sum of all the entries in the *i*-th row as the evaluating vector EV, which can be expressed as follows:
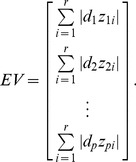
(23)


In particular, if the dimensionality of the gene data is 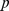, the EV has 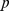 entries. After sorting the evaluating vector EV, the genes corresponding to the first 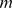 largest entries can be selected as characteristic genes.

In summary, the main steps of WPCS method are shown as follows.

1. Given the observation matrix 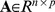, 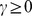, 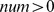.
2. To obtain the PCs 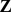, execute SPCA on the 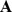.
3. To obtain the SVs, execute the SVD.
4. Obtain the WPC 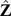 via multiplying PCs by the diagonal matrix of SVs.
5. Obtain the evaluating vector EV by summing the absolute value of WPC 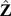 in row.
6. Sort the EV in descending order.
7. Select the genes corresponding to the first 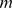 largest entries as characteristic genes.

The workflow diagram of our method is shown in Figure 5.
